# Nurses′ knowledge and practice about neonatal pain management in public hospitals in West Oromia, Ethiopia, 2022: multi-centered cross-sectional study

**DOI:** 10.1186/s12912-024-01972-3

**Published:** 2024-05-09

**Authors:** Wandimu Muche Mekonen, Addis Bilal Muhye, Mengistu Berhanu Gobeza

**Affiliations:** 1 Department of Pediatrics & Neonatal Nursing, School of Nursing and Midwifery, Institute of Health Sciences, Wollega University, Nekemte, Ethiopia; 2 Department of Pediatrics & Child Health Nursing, School of Nursing, College of Medicine and Health Sciences, University of Gondar, Gondar, Ethiopia

**Keywords:** Knowledge, Practice, Nurses, Neonatal, Pain management, Ethiopia

## Abstract

**Background:**

In low-and middle-income countries inadequate neonatal pain management persists as a significant public health issue despite the availability of guidelines. Newborns often experience pain from routine medical and surgical procedures, with limited nurses’ knowledge and suboptimal practices posing common obstacles to effective neonatal pain management in hospital settings. Hence, this study aimed to evaluate nurses’ knowledge and practices related to neonatal pain management and the factors influencing them in public hospitals in West Oromia, Ethiopia, in 2022.

**Methods:**

A multicenter cross-sectional study was conducted among 203 nurses working in public hospitals in West Oromia from 8th May-6th June 2022. Data was gathered using structured, self-administered questionnaire and sampled through simple random sampling. The collected data were coded and entered into a computer using Epi-Data version 4.6 Statistical Software. They were analyzed using the Statistical Package for Social Science (SPSS; IBM Corporation) version 26. Binary logistic regression was used to identify significant independent variables at *p* < 0.05.

**Findings:**

In this study, 127 (62.6%) exhibited adequate knowledge, while 33 (16.3%) nurses demonstrated good practice in neonatal pain management. Attending lectures or receiving training about neonatal pain management was found to be significantly associated with nurses’ knowledge of neonatal pain management [AOR, 2.31, 95%CI; 1.29–4.27]. On the other, having adequate knowledge of neonatal pain management [AOR, 3.3, 95%CI; 1.14–9.32]; the presence of a pain management policy in place [AOR, 5.44, 95% CI; 1.92–15.37] and attending lectures on neonatal pain management [AOR, 2.55, 95% CI; 1.09–5.97] were found to be significantly associated to nurses’ practices.

**Conclusion:**

Given the substandard level of nurses’ practice in neonatal pain management. It is suggested to enhance the nurses’ knowledge and practice about neonatal pain management by providing refreshment courses, training sessions, or facilitating nurses’ opportunity access to reading resources about neonatal pain management.

## Introduction

The International Association for the Study of Pain (IASP) defines pain as an unpleasant sensory and emotional experience associated with actual or potential damage [1]. It is the most disturbing and frustrating symptom experienced by hospitalized newborns [2]. Newborns are vulnerable to pain due to routine medical and surgical interventions, including vitamin K injection to prevent bleeding, heel lancets to collect blood samples, circumcision and vaccination at birth i.e. Bacillus Calmette-Guerin (BCG) and Hepatitis B (HepB) vaccine [3–8]. Similarly, newborns undergo dozens of procedures, such as suctioning, insertion of a peripheral venous catheter, mechanical ventilation and other related procedures done in the neonatal intensive care unit (NICU) that are considered as the most frequent sources of pain [9, 10]. It could be severe and potentially lead to physiological, behavioral, and cognitive abnormalities among neonates [6, 11–14]. Additionally, pain may increase morbidity and even mortality among newborns [15]. However, this neonatal pain is unrecognized because of the incorrect understanding that newborns do not experience pain and its inadequate management in hospitals, particularly in the NICU [10, 16].

The American Nurses’ Association (ANA) declared that nurses’ responsibility for pain management should involve assessing the patient’s pain, planning pharmacological and nonpharmacological pain management methods, putting the plan into action, and evaluating the patient’s reaction to the interventions [17]. Evidence shows that, an increase in nurses’ knowledge and practice can promote successful pain assessment and management among newborns [18]. However, there also are studies that uncovered gaps in nurses’ knowledge and practices regarding neonatal pain management [12, 19]. For example, a study showed that approximately two-thirds (65.6%) of the nurses did not recognize or use pain assessment tools to manage hospitalized newborns [20]. That gaps in nurses knowledge and practice remain obstacles to providing newborns quality pain management [21].

Similarly, though using clinical guidelines of pharmacological and nonpharmacological pain management methods are recommended to provide quality services, it remained underutilized by nurses in NICU [8, 22]. The reason for this is shown to be nurses’ inadequate knowledge that in turn could compromise the quality of pain management among hospitalized newborns [23, 24]. Broadly, studies show that lack of education and training on neonatal pain management, absence of pain management policy, lack of standard guidelines, and absence of analgesics in NICU do affect nurses’ knowledge and practice of neonatal pain management [25–28]. While most of studies discussed above were done elsewhere globally, nurses’ knowledge and practices related to pain management have been overlooked in sub-Saharan African countries [29]. Currently, pain assessment is considered to be a vital sign in newborns. In Ethiopia, one study done on nurses’ knowledge and practice related to pain management reported that inadequate nurses knowledge and practices of neonatal pain management [27]. It reported also limited research-based evidence regarding nurses’ knowledge and practice of neonatal pain management in the country. To address the gap, we did this study that aimed to assess nurses’ knowledge and practices and associated factors in public hospitals in West Oromia, Ethiopia in 2022. Its finding would provide a base line understanding of the current nurses’ knowledge and practices pertaining to neonatal pain management. By doing so, it indicates a crucial step toward improving pain management among neonates in NICU. This, will in turn, positively impact the quality of life of newborns and could potentially lead to decreased hospital stays.

## Methods

### Study design and setting

This institutional-based multicenter cross-sectional study was performed among nurses working at public hospitals in West Oromia, Ethiopia. There are 35 public hospitals across the West Oromia region. These hospitals are located in specific zones, including West Shewa (Ambo), Horo Guduru Wollega (Shambu), East Wollega (Nekemte), Jima (Jima), Buno Bedele (Bedele), West Wollega (Gimbi), Illubabor (Metu), and Kellem Wollega (Dembi Dolo). Each zone is at a different distance from the capital city of Ethiopia (Addis Ababa), ranging from 105 to 621 km respectively. These hospitals provide inpatient, outpatient, and referral services to the populations living in their respective zones. To obtain the number of hospitals in each zone and gather information about the study participants, we contacted the zonal health office department and the human resource management department of each hospital. These hospitals had 1931 active staff nurses, and 290 of whom worked in neonatal intensive care units (NICUs). All hospitals were purposively selected and included in this study owing to the limited sample size (NICUs). We obtained large samples (source population) from Jima University Referral Hospital (JURH) (*n* = 36), Wollega University Referral Hospital (WURH) (*n* = 21), Ambo University Referral Hospital (AURH) (*n* = 17), Nekemte Specialized Hospital (NSH) (*n* = 14), and Matu Karl Referral Hospital (MKRH) (*n* = 14). The remaining samples ranging from *n* = 4 to *n* = 11, were secured from primary and general hospitals. We subsequently allocated the samples to the site, two hundred and six (206) nurses working in neonatal intensive care units were recruited for this study from 8th May-6th June, 2022.

### Study population

The participants in this study were selected using the lottery method and provided their consent to participate. Only those working in the neonatal intensive care unit were included, while nurses who were on annual leave, maternity leave, or sick leave during the data collection period, and who had less than six months of work experience were excluded from the study.

### Sample size determination and procedure

The required sample size was determined using a single-proportion population formula. The prevalence of knowledge and practices related to neonatal pain management was 68.70% and 32.17%, respectively. This information was obtained from a study conducted at Addis Ababa public hospitals in Ethiopia [27]. We calculated the sample size using a single proportion formula for both the knowledge and practice variables. We did this using StatCal EPI INFO version 7.2 statistical software to determine the sample size for this study. To have a large sample size, we first, calculated nurses’ knowledge about neonatal pain management following the assumptions of 290 population size, 95% CI and 5% margin of error (*p* = 68.70). This yielded a sample size of 154; by adding a 10% nonresponse rate, the final sample size became 169.4≈169. Subsequently, we calculated nurses’ practices of neonatal pain management following the assumptions of 290 population size, 95% CI, and 4% margin of error (because of *p* = 32.17). This resulted in a sample size of 187; by adding a 10% nonresponse rate, the final sample size was 205.7≈206. This latter sample size was we used as a sample size for this study because it was larger than the earlier one. The number of study units from each hospital was determined using the proportional to site allocation formula:

The number of study units from each hospital were calculated as = $\frac{\text{n}\text{f}\times \text{n}\text{i}}{\text{N}}$, Where;

nf = the final sample size.

ni = number of nurses working in the neonatal intensive care unit at each hospital.

N = Total number of nurses working in the NICU at West Oromia public hospitals.

### Sampling technique and procedure

A simple random sampling technique was used to select the study subjects. During this period, the lists of the nurses were obtained from NICU head nurses. Then, based on the list obtained, a lottery method was used to select the study participants. Next, the objective of the study, confidentiality and the right to withdraw from the study at any time were briefly explained. Finally, self-administered questionnaires were distributed by data collection facilitators to those nurses who intended to participate in the study.

### Study variables

#### Dependent variables

Nurses’ Knowledge of Neonatal Pain Management.

Nurses’ Practice of Neonatal Pain Management.

### Independent variable

#### Sociodemographic factors

Age, sex, marital status, religion, education, work experience, education in neonatal pain management at university and training while in NICUs.

#### Organizational-related factors

include the presence of protocols and guidelines, the presence of analgesics, the use of standardized tools for neonatal pain scale measurements, the presence of pain management policy in place, support from leaders, provisions of in-service training, and a shortage of nurses in each work shift in the unit.

### Operational definitions

#### Adequate knowledge

Nurses scored ≥80% of the knowledge testing items for neonatal pain management [27].

#### Inadequate knowledge

Nurses scored below 80% of the knowledge testing items for neonatal pain management [27].

#### Good practice

Nurses scored ≥80% of practice testing items for neonatal pain management [27].

#### Poor practice

Nurses scored below 80% for practice testing items for neonatal pain management [27].

#### Presence of protocol/guideline

Refers to the availability of standard neonatal pain management guidelines in the neonatal intensive care unit.

#### Presence of standard tool

Availability of neonatal pain assessment tool in the neonatal intensive care unit.

### Data collection tool

The data were collected using structured, self-administered and pretested questionnaires. The tool was adopted from a previous study performed in Addis Ababa Public Hospitals (Neonatal Nurses Knowledge and Practice Survey [NNKPS]) [27]. As the tool was tested against reliability and validity in Brazil and Rwanda [30, 31], the authors declare that, it congregates the objectives of their study and also corresponding with the system in Ethiopia. The tool contains four different parts. The first part included sociodemographic characteristics such as age, sex, education level, work experience, and preservice and in-service training. The second part included twenty negatively and positively stated questions to assess the knowledge aspect. These questions were tested using True/False/I don’t know responses, which were coded as 1 = True, 2 = False, 3 = I don’t know. Then, these responses were recoded into two-point rating scale (1 = True and 0 = False) in which false and I don’t know responses were given the value 0.

The third part included practice items containing fifteen positively framed questions. These questions were tested using never/rarely/sometimes/most of the time/always responses, which were coded as 1 = never, 2 = rarely, 3 = sometimes, 4 = most of the time, and 5 = always. Then, these responses were recoded into two-point rating scale (never, rarely and sometimes = Never, whereas most of the time and always = Always). The fourth part included questions to assess factors affecting nurses’ knowledge and practices regarding neonatal pain management; these questions included questions about protocols or guidelines for pain management, the presence of analgesics in the unit, standard neonatal pain scale measurements, pain management policies, support from leadership and the shortage of nurses during each work shift.

### Data collection procedure and quality control

The pretest was performed on 5% of the sample in Adama Referral Hospital. The reliability of the tool was checked by reliability analysis, and knowledge and practice were found to be reliable tools (α = 0.787 and 0.771) respectively. We recruited one data collection facilitator for each hospital. Five BSc nurses and three health officers were supervised throughout the data collection period. The questionnaires were distributed to each hospital by data collection facilitators. All the selected nurses were given oral explanations about the purpose of the study and the time needed to complete the questionnaire (30 min), and they could withdraw from the study at any time without fear. Finally, the questionnaire was given to the nurses who provided consent and volunteered to complete and return the completed questionnaire to the data collectors.

### Data processing and analysis

The collected data were coded and entered into a computer using Epidata version 4.6 Statistical Software. The data were subsequently analyzed using the Statistical Package for Social Science (SPSS) version 26 (IBM Corporation) [32]. The negatively stated items concerning knowledge were reversed, computed, and dichotomized. The Shapiro‒Wilk test was used to determine the normality [33] of the data, and the Hosmer–Lameshow test was used to assess model fitness. The model was found to fit at P > 0.05 (knowledge = 0.761 and practice = 0.781). Descriptive statistics were used to determine knowledge and practice levels related to neonatal pain management. Categorical variables are presented as the frequency and percentage. Continuous variables are presented as the mean and standard deviation. In this study, dependent variables (knowledge and practice) were dichotomized based on the above-given operational definitions. In both domains (knowledge and practice), a total score of ≥80% was considered “adequate knowledge and good practice’, whereas a total score of <80% was considered “inadequate knowledge and poor practice’. Variables were analyzed using binary logistic regression. Variables with a *P* value < 0.25 in the bivariable analysis were included in the final multivariable analysis. Binary logistic regression was employed to assess the association between dependent and independent variables. A *p* value < 0.05 indicated a statistically significant difference in the final model.

#### Ethical approval and consent to participate

We obtained ethical clearance from the ethical review committee of the School of Nursing, University of Gondar (UoG) (Ref. no 230/2014). Then, we contacted each hospital medical director or delegated person with the original letter of permission written by the University of Gondar to each hospital to confirm permission and undertake the study. All medical directors or delegated persons readily accepted our request. Then, we were directed each neonatal intensive care unit (NICU) head nurse to cooperate with the permanent nurse staff working in the unit and as a candidate participant in the study. The confidentiality and guarantee of voluntary and anonymous participation of the nurses were maintained at all levels of the study. All the procedures were conducted based on the principles of the Helsinki Declaration.

## Results

### Socio-demographic and professional characteristics of the study participants (*n* = 203)

In this study, 206 nurses were recruited to participate, of whom 203 (98.5%) completed and returned the questionnaire. The mean±SD of nurses’ age was 29.6±3.77 years. Out of the 203 nurses, slightly half 102 (50.2%) of the nurses were male, while the majority, 153 (75.4%) of the nurses were married. The majority, 173 (85.2%) of the nurses were BSc and above nurses and slightly half 103(50.7%) of the nurses had greater than two years of NICU work experience. Furthermore, the majority 113 (55.7%) of the nurses reported that they did not attend lectures/ received training on neonatal pain management during their studies. Approximately 32.0% of the respondents received in-service training regarding neonatal pain management while in the neonatal intensive care unit (Table 1).

**Table 1:** Socio-demographic and professional characteristics of the study participants in public hospitals in West Oromia, Ethiopia, 2022 (*n* = 203)

Variables	Category	Frequency (*N*, % )
Age of participant 29±3.77(Mean±SD)	23–28	79(38.9)
	29–34	107(52.7)
	35 and above	17(8.4)
Gender of participant	Male	102(50.2)
	Female	101(49.8)
Marital status	Single	48(23.6)
	Married	153(75.4)
	Divorced	2(1.0)
Educational level	Diploma nurses	30(14.8)
	BSc and above nurses	173(85.2)
Work experience in NICU	≤2 years	100(49.3)
	>2 or above years	103(50.7)
Training on neonatal pain management at University/college	Yes	90(44.3)
	No	113(55.7)
Training on neonatal pain management being in NICU	Yes	65(32.0)
	No	138(68.0)

### Nurses’ knowledge about neonatal pain management

**Fig. 1:**
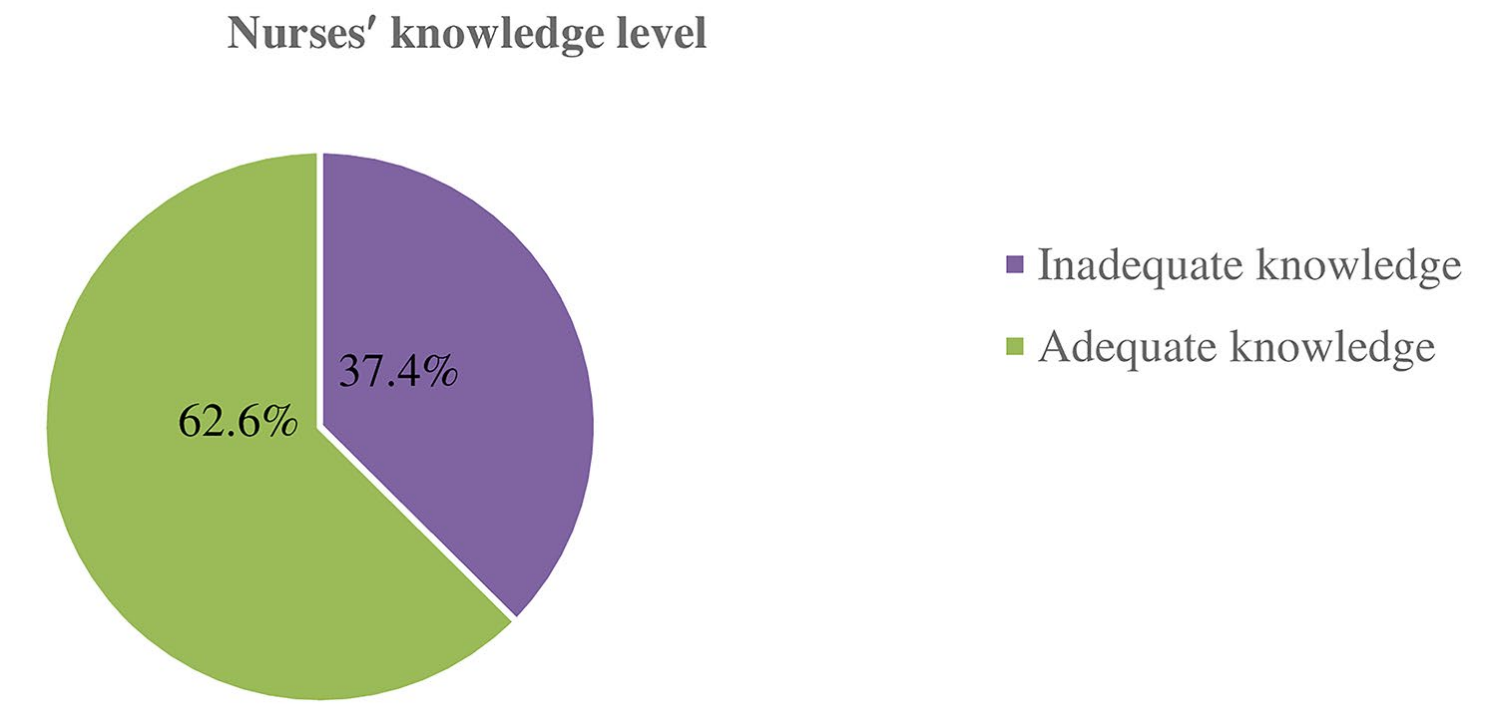
Nurses′ level of knowledge about neonatal pain management in public hospitals in West Oromia, Ethiopia, 2022 (*n* = 203)

Possessing scientific knowledge is paramount because it provides the required methods for neonatal care [34]. In the present study, the mean±SD of nurses’ knowledge was 16±3.107. The minimum and maximum knowledge items correctly answered were 4 and 20, respectively. Overall, 127 (62.6%) of the nurses had adequate knowledge about neonatal pain management (95%CI: 55.7–69.9) (Fig. 1). Among the 203 nurses, a significant proportion 165(81.3%) know that preterm newborns feel pain. Similarly, the majority of respondents 171(84.2%) recognize that full-term newborns can feel pain. Furthermore, 185 (91.1%) of the nurses were aware that pain can affect newborns’ vital signs.

The majority, 134(66%) of the nurses know the impact of light and noise on newborns’ pain reactions. Similarly, 135 (66.5%) were confident in their ability to assess neonatal pain. A total of 163 (80.3%) of the nurses identified pain as a crucial vital sign, and 155(76.4%) recognized the importance of including pain assessment in prescriptions. However, one-third of the participants 60(29.6%) were unaware of the importance of administering pain medication to newborns due to the maturity of the nervous system to feel pain (Table 2).

**Table 2:** Nurses′ knowledge about neonatal pain in public hospitals in West Oromia, Ethiopia, 2022 (*n* = 203)

Variables	Categories	
	True *N* (%)	False *N* (%)
Preterm newborns feel pain	165(81.3)	38(18.7)
Full-term newborns feel pain	171(84.2)	32(15.8)
Pain can affect a newborn’s heart rate, respiratory rate, temperature, blood pressure, oxygen saturation, and intracranial pressure.	185(91.1)	18(8.9)
Pain can affect newborns’ facial expressions, limb movements, and cries.	183(90.1)	20(9.9)
Light and noise may affect a newborn’s reactions to pain.	134(66.0)	69(34.0)
A newborn’s pain is not recognized by professionals.	23(11.3)	180(88.7)
Newborn pain is not considered by researchers.	33(16.3)	170(83.7)
Newborns react to pain in a particular way.	140(69.0)	63(31.0)
Pain is considered one of the vital signs in newborns.	163(80.3)	40(19.7)
Pain assessment in newborns must be systematized.	171(84.2)	32(15.8)
Pain assessment should be part of the nursing prescription.	155(76.4)	48(23.6)
Newborns require painkillers due to the maturity of the nervous system to feel pain.	143(70.4)	60(29.6)
Neonatal pain can be assessed without the use of scales	61(30.0)	142(70.0)
The use of scales for pain assessment is important to the practice.	179(88.2)	24(11.8)
It is important to record pain on a newborn’s chart.	188(92.6)	15(7.4)
Recording pain assessment is a prerequisite to its control	172(84.7)	31(15.3)
Nurses have sufficient knowledge to assess pain in newborns.	135(66.5)	68(33.5)
Pain management in newborns depends on its assessment	177(87.2)	26(12.8)
Recording a newborn’s pain assessment will result in relief.	156(76.8)	47(23.2)
Newborns submitted repeat painful procedures may have harmful effects on their development.	139(68.5)	64(31.5)

### Factors associated with nurses’ knowledge of neonatal pain management

Bivariable logistic regression was used to evaluate the prognostic variables. In-service training while in the NICU, educational level, lectures or training on neonatal pain management at university/college, and shortage of nurses in each work shift were associated with nurses’ knowledge of neonatal pain management (*p* < 0.25). The variables that were associated with an outcome variable were included in the multivariable logistic regression models. At *p* < 0.05, attending lectures or training about neonatal pain management at a university or college was found to be significant (*p* = 0.008). Nurses who attended lectures on neonatal pain management were more than two times more likely to have good knowledge about neonatal pain management than nurses who did not attend lectures [AOR; 2.31, 95%CI; 1.24–4.27] (Table 3).

**Table 3:** Factors associated with nurses′ knowledge toward neonatal pain management in public hospitals in West Oromia, Ethiopia, 2022(*n* = 203)

Variables	Level of knowledge		COR,95%CI	AOR,95%CI	*P*-value
	Adequate	Inadequate			
Educational level of the participants					
BSc and above Nurses	113(65.3%)	60(34.7%)	2.15(0.98,4.71)	1.59(0.66,3.56)	0.326
Diploma Nurse	14(46.7%)	16(53.3%)	1	1	
Ever attending lecture about neonatal pain management at college/university					
Yes	67(74.4%)	23(25.6%)	2.57(1.41,4.69)	2.31(1.29,4.27)	0.008*
No	60(53.1%)	53(46.9%)	1	1	
Ever attending any formal training while in NICU					
Yes	48(73.8%)	17(26.2%)	2.11(1.10,4.03)	1.75(0.89,3.42)	0.103
No	79(57.2%)	59(42.8%)	1	1	
In-service training is Provided being in NICU					
Yes	50(71.4%)	20(28.6%)	1.82(0.98,3.39)	1.52(0.80,2.91)	0.203
No	77(57.9%)	56(42.1%)	1		
Shortage of nurses in each work shift in the unit					
Yes	101(64.7%)	55(35.3%)	1.48(0.77,2.88)	1.18(0.58,2.40)	0.657
No	26(55.3%)	21(44.7%)	1	1	

### Nurses′ practice about neonatal pain management

**Fig. 2:**
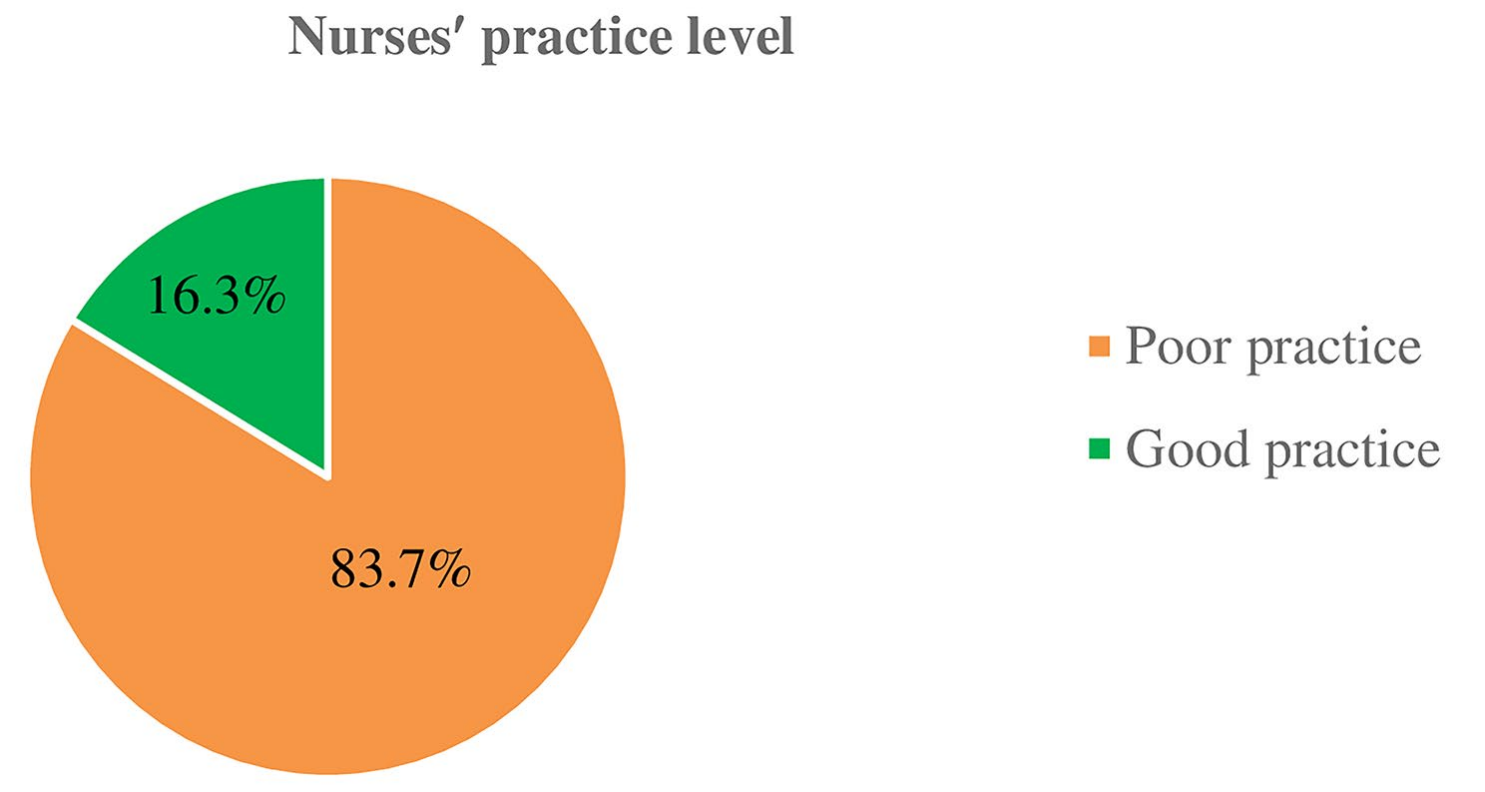
Nurses′ level of practice about neonatal pain management in public hospitals in West Oromia, Ethiopia, 2022 (*n* = 203)

The mean±SD of nurses’ practice was 7.79±3.616. The minimum and maximum numbers of correctly answered practice items were 0 and 15, respectively. Overall, the findings indicated that, only 33 (16.3%) of the nurses had good practices for neonatal pain management (95%CI: 11.8–23.1) (Fig. 2). The majority 123(60.6%) of the respondents always assessed newborns through crying, followed by 103 (50.7%) through facial expression. Nearly half 107 (52.7%) of the nurses used the pain scale to assess neonatal pain, whereas 96 (47.3%) did not use it. Similarly, half 102 (50.2%) of the nurses documented the assessed newborn pain on medical charts. Regarding nonpharmacological pain management, 68 (33.5%) of the nurses used nonnutritive suckling, and 136 (67%) of the nurses encouraged breastfeeding to relieve pain in newborns. Furthermore, the majority 120 (59.1%) of the nurses always used skin-to-skin contact, 109 (53.7%) used more than one nonpharmacological approach, and 139 (68.5) used both pharmacological and nonpharmacological management to relieve pain in the newborns (Table 4).

**Table 4:** Nurses′ practice about neonatal pain management in public hospitals in West Oromia, Ethiopia 2022 (*n* = 203)

Variables	Categories	
	Never *N* (%)	Always *N* (%)
I assess newborns’ pain through crying.	80(39.4)	123(60.6)
I assess newborns’ pain through facial expressions.	100(49.3)	103(50.7)
I assess newborns’ pain through body movement and agitations.	106(52.2)	97(47.8)
I assess newborns’ pain through their vital signs.	73(36.0)	130(64.0)
I use scales to assess pain in newborns.	96(47.3)	107(52.7)
I record newborns’ pain scores on their medical charts.	101(49.8)	102(50.2)
I use non-nutritive suckling to relieve pain in newborns.	135(66.5)	68(33.5)
I encourage breastfeeding to relieve the pain in the newborn.	67(33.0)	136(67.0)
I encourage skin-to-skin contact to relieve pain in newborns.	83(40.9)	120(59.1)
I offer oral glucose or sucrose to relieve newborn pain before painful procedures.	158(77.8)	45(22.2)
I offer oral glucose or sucrose to relieve newborn pain during painful procedures.	140(69.0)	63(31.0)
I position the newborn to relieve their pain.	69(34.0)	134(66.0)
I perform facilitated tucking in newborns during pain full procedures.	113(55.7)	90(44.3)
I use more than one non-pharmacological measurement to relieve the pain of the newborns.	94(46.3)	109(53.7)
I use pharmacological and non-pharmacological combined to relieve pain in newborns.	64(31.5)	139(68.5)

### Factors associated with nurses′ practices of neonatal pain management

Bivariable logistic regression was performed, and having adequate knowledge about neonatal pain management, sex, attending lectures on neonatal pain management at the university or college, in-service training, availability of protocols and pain management guidelines, support from leaders, use of standard neonatal pain scale measurements, and the presence of a pain management policy in place were found to be significant (*p* < 0.25). By controlling for the effect of confounding variables, multivariable logistic regression was performed. Variables such as attending lectures/training on neonatal pain management at university/college, having adequate knowledge and the presence of a pain management policy in place were found to be significant at *p* < 0.05.

Nurses who attended lectures on neonatal pain management were more than two times more likely to practice good neonatal pain management than were those who did not attend [AOR 2.55; 95%CI; 1.09–5.97]. Nurses who had adequate knowledge of neonatal pain management were more than three times more likely to practice neonatal pain management well than nurses with inadequate knowledge [AOR, 3.26, 95% CI; 1.14–9.32; (*r* = 0.457, P = < 0.001)]. Nurses who had pain management policies in place were more than five times more likely to practice good neonatal pain management than were those who did not have a pain management policy [AOR, 5.44, 95%CI 1.92–15.37](Table 5).

**Table 5:** Factors associated with nurses′ practice toward neonatal pain management in public hospitals in West Oromia, Ethiopia, 2022(*n* = 203)

Variables	Level of practice		COR, 95%CI	AOR,95%CI	*P*- value
	Good	Poor			
Knowledge level					
Adequate Inadequate	28(22.0%)	99(78.0%)	4.02(1.48,10.91)	3.26 (1.14,9.32)	0.028*
	5(6.6%)	71(93.4%)	1	1	
Sex of participant					
Female Male	24(23.8%)	77(76.2%)	3.22(1.41,7.34)	2.38 (0.98,5.79)	0.056
	9(8.8%)	93(91.2%)	1	1	
Pre-service training					
Yes No	22(24.4%)	68(75.6%)	3.00(1.37,6.59)	2.55 (1.09,5.97)	0.032 *
	11(9.7%)	102(90.3%)	1	1	
Presence of guidelines					
Yes No	19(21.8%)	68(78.2%)	2.04(0.96,4.33)	0.99(0.35,2.82)	0.993
	14(12.1%)	102(87.9%)	1	1	
Presence of standard neonatal pain scale measurement					
Yes No	23(28.0%)	59(72.0%)	4.33(1.93,9.7)	2.06(0.67,6.08)	0.193
	10(8.3%)	111(91.7%)	1	1	
Pain management policy					
Yes No	28(26.4%)	78(73.6%)	6.61(2.43,17.9)	5.4(1.92,15.37)	0.001*
	5(5.2%)	92(94.8%)	1	1	
Support from leadership					
Yes No	19(22.4%)	66(77.6%)	2.14(1.00,4.56)	1.75(0.73,4.19)	0.207
	14(11.9%)	104(88.1%)	1	1	
In-service training is provided					
Yes No	15(21.4%)	55(78.6%)	1.74(0.82,3.71)	1.16(0.49,2.74)	0.735
	18(13.5%)	115(86.5%)	1	1	

## Discussion

The present study assessed nurses’ knowledge and practices of neonatal pain management and associated factors in public hospitals in West Oromia, Ethiopia. This study showed that 62.56% (95% CI: 55.7–69.9) of the nurses had adequate knowledge of neonatal pain management. These findings were greater than those of a study conducted in Rwanda (25.8%) [35]. The observed discrepancy could be attributed to the differences in the study populations and study units. The study conducted in Rwanda considered nurses working other than neonatal intensive care units, such as pediatric units, to assess nurses’ knowledge about neonatal pain management, which could affect nurses’ knowledge due to lack of exposure at the site. This finding is consistent with that of a study conducted in Gambia, which reported that working units were significantly associated with nurses’ knowledge [36]. Another possible explanation could be that, unlike in the current study, which involved lectures on neonatal pain management, a significant proportion of the nurses in Rwanda did not attend course training or lectures about neonatal pain management. These findings are consistent with those of studies conducted in Ghana (61.1%), the University of Gondar Comprehensive Specialized Hospital (67.9%), and Addis Ababa City Public Hospital (68.7%) [19, 27, 37].

Thus, the consistency of this study is justified. This may have been due to the same study unit and exclusion criteria. The current study highlighted neonatal intensive care unit nurses and nurses with more than six months of work experience, which is similar to the findings of other studies. Having more working experience and being consistent with a particular study unit were significantly and positively associated with nurses’ knowledge of pain management in children. This study was supported by a study conducted at the University of Gondar Specialized Hospital, and Edward Francis Small Teaching Hospital, in Gambia [36, 37].

This study indicated that nurses’ knowledge of neonatal pain management was positively associated with attending lectures on neonatal pain management at university/college (*p* = 0.008). Nurses who attended lectures on neonatal pain management were more than two times more likely to have adequate knowledge about neonatal pain management than nurses who did not attend [AOR, 2.31; 95%CI = 1.29–4.27]. This finding is consistent with that of a study conducted in Turkey and Addis Ababa public hospitals [27, 38]. This finding suggested that providing preservice or in-service training in neonatal pain management will improve nurses’ knowledge of pain management in hospitalized newborns.

The current study also revealed that 33(16.3%) (95% CI: 11.8–23.1) of the nurses had good practices regarding neonatal pain management. This finding is consistent with a study conducted in Rwanda (15.2%) [35]. This might be due to a lack of adequate knowledge about neonatal pain management in both study areas. This was supported by a study conducted in Addis Ababa public hospitals, which suggested that possessing adequate knowledge is positively correlated with the practice of neonatal pain management [27]. However, this percentage is lower than that reported in studies in Malaysia (41.67%), Ghana (57.8%), Addis Ababa public hospitals (32.2%) and the West Shoa zone, Ethiopia (37.3%) [18, 19, 27, 39]. The perceived discrepancy could be due to the absence or poor quality of neonatal pain management courses in nursing, limitations in training/refreshing staff nurses about neonatal pain management and a lack of resources such as updated guidelines for managing neonatal pain. Furthermore, the difference might be due to the lack of established pain management policies in some hospitals [40, 41].

The logistic regression model also identified significant variables associated with nurses’ practice of neonatal pain management. Nurses who had a pain management policy in place were more than five times more likely to demonstrate good practice of neonatal pain management than were those who did not have a pain management policy in place [AOR, 5.44 95%CI; 1.92–15.37, *P* = 0.001)]. This finding is consistent with that of a previous study conducted in Addis Ababa public hospitals [27]. This indicates that the implementation of a pain management policy in each unit is crucial for improving neonatal pain management, which is supported by previous study [27]. Unlikely, a qualitative study conducted in Iran reported that lack of pain management policy were negatively associated with nurses practice of neonatal pain management [41]. Therefore presence of pain management policy in hospital setting, particularly in neonatal intensive care unit will assist the nurses to adhere in good practice of neonatal pain management.

Nurses who attended lectures on neonatal pain management at university/college were more than twice as likely to practice neonatal pain management than were those who did not attend lectures on neonatal pain management [AOR, 2.55, 95%CI; 1.29–4.27, *P* = 0.032)]. This finding is similar to that of a study conducted in the Rwanda and Addis Ababa public hospitals [27, 35]. However, unlike this study, the study conducted in Iran reported that lack of educational courses about neonatal pain management were the main obstacles to implement pain management in newborns [41]. This indicates that presence of gap and poor delivery of educational courses pertaining to neonatal pain management at University/College. Similarly, nurses with adequate knowledge of neonatal pain management were more than three times more likely to practice neonatal pain management than were those with inadequate knowledge [AOR; 3.26, 95% CI; 1.14–9.32, *P* = 0.028)].

This positive association was also found in a study conducted in Thailand, Rwanda and Addis Ababa public hospitals, which reported that possessing adequate knowledge was significantly and positively associated with nurses’ practices of neonatal pain management [27, 35, 40]. This finding suggests that, to be distinctive in neonatal pain management, nurses’ competencies should be promoted by providing training about neonatal pain management and providing guidelines for neonatal pain management in the units. Pearson’s correlation coefficient test was examined to see the association between knowledge and practice in the context of neonatal pain management. The results indicated a statistically significant positive correlation between knowledge and practice (*r* = 0.203, *p* = 0.004*), suggesting that the greater the knowledge of nurses about neonatal pain management is, the greater the probability of good practices of neonatal pain management. However, the self-reported nature of the data may limit the generalizability of the findings to clinical practice.

## Conclusion

Nurses′ knowledge and practice about neonatal pain management was low in the study area in relation to the previous studies. Multivariable logistic regression analysis revealed that attending lectures on neonatal pain management at university/college was significantly and positively associated with nurses’ knowledge of neonatal pain management. Attending lectures on neonatal pain management, having adequate knowledge about neonatal pain management, and the presence of a pain management policy in place were factors significantly and positively associated with nurses’ practices of neonatal pain management. In addition, the present findings offer significant evidence to healthcare providers regarding pain in newborns. Moreover, this study provides basic information for hospitals to focus on how to improve nurses’ knowledge and practices regarding neonatal pain management. We suggest that education and training in neonatal pain management should be considered when addressing this problem. The ministry of Health and Nursing College should review the educational curriculum to improve neonatal pain management. Moreover, a pain management policy should be established for all hospitals in the NICU. Finally, we recommend that researchers willing to conduct the same study support a qualitative study.

### Limitations

This study has several limitations. Although neonatal pain management is critical, nurses’ knowledge about and practices related to its management were not adequately assessed in Ethiopia. Hence, the authors were challenged to compare the present study with the previous evidence. However, we believe that this study could address this problem by providing a baseline for other researchers who are willing to conduct related studies. Moreover, due to the nature of the data collection method, (i.e., self-reported) data, the results may not indicate actual clinical practice.

## Data Availability

The authors can provide the data sets used for the current study upon request.

## Abbreviations

AOR: Adjusted odd ratio
BCG: Bacillus Calmette-Guerin
Hep B: Hepatitis B vaccine
IASP: International Associations for the Study of Pain
IRB: Institutional Review Board
NICU: Neonatal Intensive Care Unit
r: Spearman correlation coefficient
SPSS: Statistical Package for Social Science
UoG: University of Gondar
